# Morphology of third mandibular molars – an in vivo cone beam computed tomography study in Lower Silesia

**DOI:** 10.1186/s12903-026-09559-x

**Published:** 2026-08-13

**Authors:** Anna Olczyk, Barbara Malicka, Paweł Stenzel, Urszula Sękowska, Katarzyna Skośkiewicz-Malinowska

**Affiliations:** 1 Department of Conservative Dentistry With Endodontics, Wroclaw Medical University, Krakowska 26, 50-425 Wrocław, Poland; 2 Conservative Dentistry With Endodntics Clinic, University Dental Center, Krakowska 26, 50-425 Wrocław, Poland

**Keywords:** Molar, Third, Mandible, Cone-Beam Computed Tomography, Dental Pulp Cavity, Sex Factor

## Abstract

**Background:**

Understanding the root and canal morphology of mandibular third molars (MTMs) is crucial for anatomical characterization of human dentition and effective endodontic treatment. This study investigates the number of roots, canal configurations, and the prevalence of C-shaped canals in MTMs using Cone Beam Computed Tomography (CBCT).

**Objectives:**

To analyse root number, sex-related differences, canal configurations, and the prevalence of C-shaped canals in MTMs.

**Methods:**

This cross-sectional study analysed CBCT scans of 261 MTMs from patients aged 18 and over according to Vertucci’s classification. Data were collected at the X-Ray Diagnostic Laboratory of Wrocław Medical University from January to April 2022. Statistical analyses included chi-squared tests with Bonferroni correction (*p* < 0.05). Effect size was assessed using Cohen’s “w” (0.172) with test power of 0.64 at an alpha level of 0.05.

**Results:**

Most MTMs had two roots (79.69%), followed by three roots (11.81%) and a single root (8.43%). Two-rooted teeth were more frequent in males (87.27%) than in females (74.17%, *p* = 0.027). Type I was the predominant root canal configuration in all categories. C-shaped canals were present in 5.36% of teeth, predominantly in single-rooted teeth (71.43%) and females (92.86%). Interobserver agreement was excellent (κ = 0.83), with an overall agreement of 91.7%. Intraobserver agreement was κ = 0.84 and κ = 0.86.

**Conclusions:**

This study highlights variations in anatomical diversity of MTMs, emphasizing clinical importance of CBCT for accurate diagnosis and treatment planning, expanding anatomical knowledge. Findings underscore the prevalence of C-shaped canals and sex-related differences in root structure.

**Supplementary Information:**

The online version contains supplementary material available at https://doi.org/10.1186/s12903-026-09559-x.

## Introduction

Mandibular third molars (MTMs), commonly referred to as wisdom teeth, exhibit significant anatomical variability, posing challenges for dental practitioners in endodontic and surgical procedures. Accurate knowledge of root canal morphology is essential for effective treatment planning and achieving successful clinical outcomes.

Although numerous studies have investigated the anatomy of mandibular first (Al-Habib et al. 2024) and second molars (Yang et al. 2022), research focusing specifically on mandibular third molars remains limited (Al-Qudah et al. 2023). To date, only one study has investigated MTMs in the Polish population (Tomaszewska et al. 2018b).

Understanding the anatomical complexity of MTMs is critical for successful endodontic treatment. Beyond their functional role, MTMs can also serve important purposes when opposing teeth are absent, such as acting as fixed prosthetic abutments, providing support for partial dentures, or being used for autotransplantation (De Bruyn et al. 2020). Moreover, the presence of mandibular third molars prevents overeruption of opposing maxillary third molars, which can occur in the absence of antagonist (Alyami 2023). In some cases, endodontic treatment of mandibular third molars may represent a more conservative alternative to extraction, particularly when surgical removal is associated with increased risks, e.g. injury to the inferior alveolar nerve (Putrino et al. 2025).

Current research on root canal configurations on MTMs reveals several gaps. Many studies lack population diversity, which limits the generalizability of findings to other groups. It should be noted that most available studies focus on Asian and Middle Eastern populations, while data on Central European populations remain scarce. Furthermore, few studies examine root number, canal configuration, and C-shaped morphology in a single study, limiting the clinical relevance of existing findings (Ahmad et al. 2016; Somasundaram 2017; Tomaszewska et al. 2018b; Singh et al. 2020; Priyank et al. 2023). The absence of standardized terminology and classification systems makes direct comparisons across studies difficult. Inconsistencies in data presentation, limited statistical analyses, small sample sizes, and insufficient stratification further reduce the reliability and applicability of existing findings.

Cone-beam computed tomography (CBCT) allows for three-dimnesional assessment of root canal morphology with relevant clinical accuracy, as well as it is widely used in in vivo anatomical studies (Neelakantan et al. 2010; Naseri et al. 2019). While micro-computed tomography (micro-CT) offers higher resolution, it could not be used in vivo due to high radiation exposure (Ahmed 2022). The objective of this study was to thoroughly evaluate the morphology of the mandibular third molar based on CBCT in a Central European population. Specifically, the study aimed to determine the prevalence of different root numbers, analyse root canal configuration according to the Vertucci classification, assess the prevalence of C-shaped root canal systems, and evaluate potential sex differences. Furthermore, the study aimed to investigate clinically relevant relationships between root number and canal morphology in order to improve anatomical prediction in endodontic practice.

To our knowledge, this is one of the few CBCT-based studies evaluating root number, root canal configuration, sex-related differences, and C-shaped canal prevalence simultaneously in mandibular third molars from a Central European population.

## Material and methods

### Data collection

The study was designed and reported in accordance with the STROBE (Strengthening the Reporting of Observational Studies in Epidemiology) guidelines (von Elm et al. 2008). The CBCT scans were obtained from the Academic Dental Polyclinic of Wrocław Medical University in Poland, specifically from the X-Ray Diagnostic Laboratory. These scans were conducted for diagnostic or other dental purposes, e.g. implants, orthodonitcs, surgical evaluation of impacted teeth or endodontic diagnosis, between January and April 2022 using a Rayscan α-Plus 130 (Rayscan 700) CBCT scanner (Ray Europe GmbH, Seoul, South Korea). The scanner's specifications included a field of view ranging from 3 × 4 cm to 13 × 10 cm, with settings of 60–90 kV, 4–17 mA, a scan duration of 14 s, and voxel sizes of 70 µm, 160 µm, 200 µm, or 280 µm. The slice thickness varied from 0.07 mm to 3 mm.

To standardize the study, only full-mouth CBCT scans with a voxel size of 160 µm, slice thickness of 0.16 mm, field of view of 13 × 10 cm, and settings of 90 kV and 4 mA were used in final analysis. Scans with other voxel sizes were excluded to minimize differences in image resolution. Data were reviewed using Rayscan SMART Dent software (Ray Co., Ltd, Seoul, South Korea) and OnDemand3DApp software (Cyber Med, Seoul, South Korea). This standardization was done to improve the comparability of anatomical observations across all examined scans and minimize methodological diversity in methodological approach. Research access to the data was granted on June 7, 2022. Study approval was obtained from the Bioethics Committee of Wrocław Medical University (approval no. KB 206/2022), in accordance with the Declaration of Helsinki. Informed consent was obtained, and data were anonymized to prevent participant identification. All CBCT datasets were provided in DICOM format without directly identifiable personal information (names and surnames). Each data set was assigned a unique study code. Only limited demographic data, such as sex, date of birth, and study date, were retained for analysis.

The images were evaluated by two endodontists with 5 and 30 years of professional experience, respectively. A Beacon Display C25W screen (Shenzhen Beacon Display Technology Co., Ltd.) was used for analysis, featuring a resolution of 1920 × 1080 pixels. Various image adjustments, including zoom, magnification, brightness, and contrast, were applied as needed. Assessments were performed in a dimly lit room, with no more than 15 scans were evaluated per session to minimize fatigue.

Before evaluating the CBCT scans, both endodontists underwent training and calibration using 36 sample CBCT scans (approximately 10% of the total sample) representing the eight types of Vertucci's classification. The calibration set included examples of all root canal configurations identified in the study sample and was designed in accordance with previously published CBCT-based studies assessing interobserver reliability in root canal morphology (Mokhtari et al. 2016). Interobserver agreement was excellent (κ = 0.83), with an overall agreement of 91.7%, according to the interpretation criteria proposed by McHugh (2012) (McHugh 2012). To assess intraobserver reliability, the same 36 scans after two weeks were re-evaluated independently in random order and without access to initial results. Calculation of Cohen’s kappa coefficient, yielded high intraobserver agreement for both examiners: A (κ = 0.86); B (κ = 0.84). After calibration all CBCT scans (n = 316) were independently evaluated by one examiner and subsequently by a second more experienced dentist. Any discrepancies between the two observers were resolved by consensus.

Inclusion criteria were: participants aged 18 or older (continuous variable, with no further categorization); teeth without caries, fillings, prosthetic crowns, or post-core inlays; teeth without previous endodontic treatment; teeth with closed apices, free from resorptions or calcifications; and artifact-free CBCT images. Tooth rotation (mesiolingual or distolingual) did not disqualify the tooth from the study.

Exclusion criteria: teeth with caries, fillings, prosthetic crowns, or post-core inlays; teeth with open apices, resorptions, or calcifications; and CBCT images with artifacts. Tooth rotation (mesiolingual or distolingual) was not considered an exclusion criterion.

**Fig. 1:**
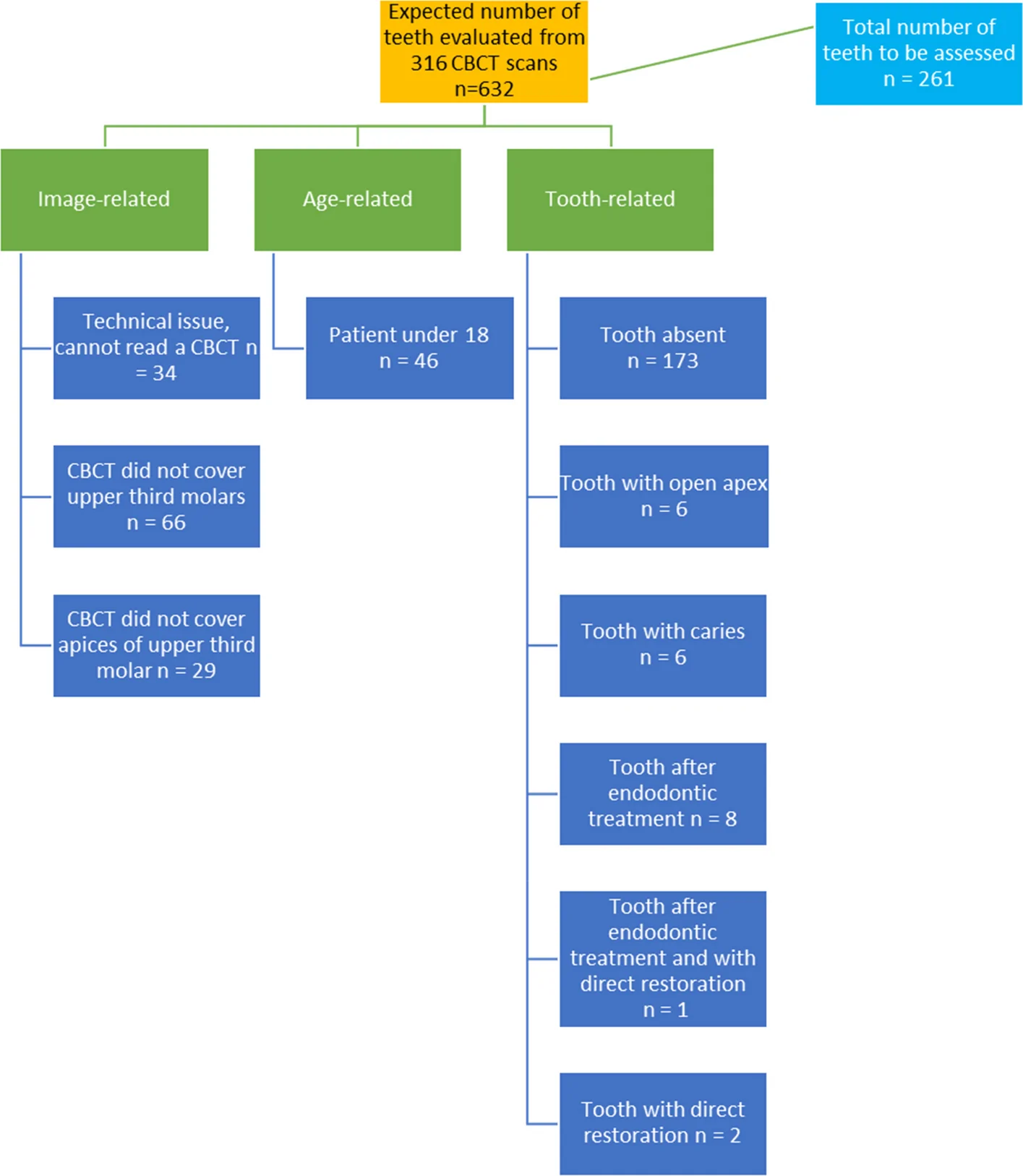
Exclusion criteria (STROBE Flowchart)

Following the analysis of 316 CBCT scans from a potential total of 632 teeth, 261 mandibular third molars met the inclusion criteria. The main reasons for exclusion were tooth absence (n = 173), incomplete imaging coverage of the region of interest, and patient age below 18 years (n = 46). Additional exclusions included teeth with open apices, caries, previous endodontic treatment, or restorations. Figure 1 shows the inclusion and exclusion criteria for the study.

The number of roots, root canals, and root canal configurations were assessed according to Vertucci's classification (1974) (Vertucci et al. 1974), which categorizes root canal morphology into eight distinct types (I-VIII) based on the number and configuration of canals from the pulp chamber to the apex, representing specific patterns of how canals may branch, merge, or remain separate throughout the root. Root and root canal number were treated as categorical variables.

The most widely used and clinically recognized method for describing root canal morphology is the Vertucci classification, which is why it was chosen for our study, although modifications and alternative classification systems have been proposed to address more complex canal anatomies (Ahmed et al. 2017; Al-Qudah et al. 2023; Gulabivala and Ng 2023). Direct comparison with previously published data is possible thanks to its widespread use in CBCT-based anatomical studies. Also, all observed canal configurations in the analysed sample could be classified accordingly within this classification. Therefore, the use of more complex classification systems would not have provided additional clinically relevant information for the present dataset.

Vertucci's classification describes root canal configurations as follows:

- Type I – single canal from the pulp chamber to the apex of the root
- Type II – two canals exit the pulp chamber and join into one canal to the apex of the root
- Type III – single canal leaves the pulp to divide into two and then join again and finish at the apex of the root
- Type IV – two canals leave the pulp chamber and two canals continue to the apex
- Type V – single canal leaves the pulp chamber and divides into two canals that continue to the apex
- Type VI – two canals leave the pulp chamber, then merge into one and divide into two again and finish as to canals to the apex
- Type VII – single canal leaves the pulp chamber, then divides into two, then merges into one to finally divide into two canals and finish as two canals at the apex
- Type VIII – three canals leave the pulp chamber, and three canals exit to the apex.

This cross-sectional study investigated the root canal morphology of mandibular third molars (MTMs) in a population from Lower Silesia using CBCT imaging. The analysis included the assessment of number of roots, root canal configurations according to Vertucci’s classification, prevalence of C-shaped canals, sex-related differences in root number. The findings were further compared with previously published data.

### Statistical analysis

Data analysis was conducted by the Statistical Analysis Center of Wroclaw Medical University using Statistica 13 (2017) software (TIBCO Software Inc., USA). The analysis included the number of roots (Table 1), the number of roots by sex (Table 2, Table 3), and Vertucci’s classification (Tables 4 and 5). Table 2 presents chi-squared and p-values comparing the frequency of each tooth type by sex. The chi-squared test yielded a significance level below 0.05 (Table 2). The Bonferroni correction was applied to control for Type I error, adjusting for incidental findings of significant differences. The threshold for statistical significance was set at 0.05. 95% Confidence intervals for the observed rates are provided in Tables 1 and 6. All CBCT scans obtained between January and April 2022, after meeting the inclusion criteria, were included in the study. Therefore, the sample size was determined based on data availability rather than a priori calculations, due to the retrospective design of the study. Results should be interpreted as exploratory. All available scans (*n* = 316) were analysed, and the power of the analysis for mandibular third molars meeting the inclusion criteria (*n* = 261) was calculated afterwards using the “power.chisq.test” function from the “DescTools” R package. A post hoc power analysis of chi-squared test on 2 × 4 tables with sample size of 261 demonstrated that the available sample provided 64% power to detect a Cohen’s effect size of w = 0.172, with an alpha level of 0.05 (two-tailed test).

**Table 1:** Distribution (%) of roots in mandibular third molars with 95% confidence intervals (CI)

Number of roots	n (%)	Proportion (95% CI)
1	22 (8.43)	0.08 (0.05- 0.12)
2	208 (79.69)	0.8 (0.74- 0.84)
3	31 (11.88)	0.12 (0.08- 0.16)
Total number	261 (100)	-

**Table 2:** Distribution (%) of roots in mandibular third molars based on sex

Number of roots	Female n (%)	Male n (%)	Chi-square statistic	*P*-value
1	18 (11.92)	4 (3.64)	7.8	**0.02**
2	112 (74.17)	96 (87.27)		
3	21 (13.91)	10 (9.09)		
Total number	151 (100)	110 (100)		
*Statistical significance threshold set at p* < *0.05*

**Table 3:** A post hoc test of the differences in the frequency of roots between the sexes (females vs. males) in mandibular third molars with Bonferroni correction

Comparison	Chi-square statistic	*P*-value
**1-root vs. others**	5.96	0.051
**2-root vs. others**	6.57	**0.027**
**3-root vs. others**	1.51	0.705
Statistical significance threshold set at *p* < 0.05

**Table 4:** Vertucci's canal distribution in the studied population

		**Vertucci Type**						
**Tooth root type**	**Root**	**Type I n (%)**	**Type II n (%)**	**Type III n (%)**	**Type IV n (%)**	**Type V n (%)**	**Type VI n (%)**	**Type VIII** **n (%)**
**Single-rooted teeth** **(S)**	S	9 (40.91)	2 (9.09)	0	7 (31.82)	1 (4.55)	0	3 (13.64)
	Female/Male	6/3	2/0	0/0	6/1	1/0	0	3/0
**Two-rooted teeth** **(M, D)**	M	133 (64.42)	8 (3.85)	2 (0.96)	62 (29.81)	3 (1.44)	0	0
	Female/Male	80/53	4/4	1/1	25/37	2/1	0/0	0/0
	D	197 (94.71)	4 (1.92)	0	5 (2.40)	0	1 (0.48)	1 (0.48)
	Female/Male	106/91	3/1	0/0	2/3	0/0	1/0	0/1
**Three-rooted teeth** **(MB, ML, D)**	MB	23 (100)	0	0	0	0	0	0
	Female/Male	14/9	0/0	0/0	0/0	0/0	0/0	0/0
	ML	23 (100)	0	0	0	0	0	0
	Female/Male	14/9	0/0	0/0	0/0	0/0	0/0	0/0
	D	22 (95.65)	0	0	1 (4.35)	0	0	0
	Female/Male	13/9	0/0	0/0	1/0	0/0	0/0	0/0
**Three-rooted teeth** **(M, DB, DL)**	M	6 (75.00)	0	0	2 (25.00)	0	0	0
	Female/Male	5/1	0/0	0/0	2/0	0/0	0/0	0/0
	DB	8 (100)	0	0	0	0	0	0
	Female/Male	7/1	0/0	0/0	0/0	0/0	0/0	0/0
	DL	7 (87.5)	0	0	0	1 (12.5)	0	0
	Female/Male	6/1	0/0	0/0	0/0	1/0	0/0	0/0

**Table 5:** Percentages (in relation to the total number of canal types) along with their 95% confidence intervals (CI)

Number of roots	Root	Total number	Vertucci’s canal types					
			Type I Proportion (95% CI)	Type II Proportion (95% CI)	Type III Proportion (95% CI)	Type IV Proportion (95% CI)	Type V Proportion (95% CI)	Type VIII Proportion (95% CI)
1	S	22	0.41 (0.207—0.637)	0.09 (0.011—0.292)	-	0.32 (0.139—0.549)	0.05 (0.001 -0.228)	0.14 (0.029—0.349)
2	M	208	0.64 (0.570—0.705)	0.04 (0.017—0.074)	0.01 (0.001—0.034)	0.30 (0.237—0.365)	0.01 (0.001—0.034)	-
	D	208	0.95 (0.913—0.978)	0.02 (0.005—0.049)	-	0.02 (0.005—0.049)	-	-
3	MB	23	1	-	-	-	-	-
	ML	23	1	-	-	-	-	-
	D	23	0.96 (0.781—0.999)	-	-	0.04 (0.001—0.220)	-	-
3	M	8	0.75 (0.349—0.9681)	-	-	0.25 (0.032—0.651)	-	-
	DB	8	1	- -	- -	- -	- -	- -
	DL	8	0.88 (0.474—0.997)	-	-	-	0.13 (0.003—0.527)	-

Control for confounding was addressed by inclusion criteria. Only artifact-free CBCT images were included; as a result, no missing data were present in the analyses. All available scans meeting the inclusion criteria were analysed; no complex sampling strategy was applied, and hence no analytical methods to account for sampling design were required. Sensitivity analyses or interaction analyses were not applicable in this study. Only unadjusted estimates were presented. No confounder-adjusted analyses were performed beyond restriction by inclusion criteria. Age was recorded as a continuous variable, no age-based subgroup analysis was done, all included teeth had closed apices. Relative and absolute risk estimates were not applicable in this morphological study. Subgroup analyses were performed by sex for the number of roots and root canal configurations. Post hoc comparisons with Bonferroni correction were conducted to assess differences between male and female participants.

The study adhered to the guidelines outlined in the Anatomical Quality Assurance (AQUA) checklist (Tomaszewski et al. 2017) attached in supplement 1.

## Results

### Observer agreement

Calibration phase showed excellent reliability with interobserver agreement assessment with Cohen’s κ = 0.83, with an overall agreement of 91.7%. Intraobserver agreement was also high, with Cohen’s kappa values of 0.84 and 0.86 for examiners A and B, respectively.

### Characteristics of the study population

From 261 mandibular third molars analysed in the study, from 163 patients, 151 belonged to females (57.9%) and 110 males (42.1%). The mean age of the patients was 33.95 ± 12.23 years, with a median of 29.86 years, with minimum of 18.01 and maximum of 70.9 years of age. 126 teeth were located on the left side and 135 on the right side. 65 (39.9%) patients presented with a single mandibular third molar, while 98 (60.1%) had bilateral mandibular third molars.

### Root count (overall)

Out of the 261 teeth scans analysed, two-rooted teeth were the most prevalent, accounting for 79.69% (*n* = 208). Three-rooted teeth were less common, at 11.81% (*n* = 31), and single-rooted teeth were the least common, making up 8.43% (*n* = 22) (see Table 1).

### Root count by sex

The frequency of occurrence of various mandibular third molar types, categorized by the number of roots, was also evaluated based on patient sex (Tables 2, and 3).

In all chi-square test indicated a statistically significant connection between sex and root number distribution (*p* = 0.02). However, post hoc comparisons showed that only the proportion of two-rooted teeth differed significantly between sexes (112 teeth, 74.17% vs 96 teeth, 87.27%) after Bonferroni correction (*p* = 0.027). Single-rooted teeth were more frequent in females than in males (18 teeth, 11.92% vs. 4 teeth, 3.64%), but this difference did not reach statistical significance (*p* = 0.051). Three-rooted teeth were also more frequent in females than in males (21 teeth, 13.91% vs. 10 teeth, 9.09%), but the difference was not statistically significant (*p* = 0.705).

### Distribution of root canal configurations according to root morphology

Table 4 illustrates the distribution of Vertucci canal configurations across the different root morphologies observed in this study, corresponding 95% confidence intervals are shown in Table 5. Overall, Vertucci Type I was the most prevalent canal configuration (80.6%), followed by Type IV (14.5%), Type II (2.6%), Type V (0.9%), Type VIII (0.8%), Type III (0.4%), and Type VI (0.2%). The chart also reveals a higher frequency of two canals (corresponding to Type IV configurations) in the mesial roots (29.81%) compared to distal roots (2.40%).

Single-rooted teeth predominantly exhibited Type I root canals according to Vertucci’s classification, with 9 teeth (40.91%) identified as such. Type IV canals were less common, observed in 7 teeth (31.82%). Types III, VI, and VII were absent from the study group, while Type VIII (3 teeth, 13.64%) was found infrequently. Similarly, Types II (2 teeth, 9.09%) and V (1 tooth, 4.55%) were observed sporadically.

In two-rooted mandibular third molars, Vertucci’s Type I root canal configuration was the most frequently observed in both mesial (133 teeth, 64.42%) and distal (197 teeth, 94.71%) roots. Type IV was also relatively common in the mesial roots, occurring in 62 teeth (29.81%). Types II (8 teeth, 3.85%), III (2 teeth, 0.96%), and V (3 teeth, 1.44%) were also identified. In distal roots, Types II (4 teeth, 1.92%), IV (5 teeth, 2.40%), VI (1 tooth, 0.48%), and VIII (1 tooth, 0.48%) appeared sporadically. Among all the teeth examined, there were only 2 cases of fused roots, and those were among 2 rooted teeth. Two cases of fused roots were observed in two-rooted mandibular third molars, both in female patients, exhibiting Type I canal configurations in distal roots and Type I or Type V configurations in mesial roots. Because of the very low frequency, no further statistical analysis of fused roots was performed.

Three-rooted teeth exhibited two root configurations. In the first, roots were mesiobuccal (MB), mesiolingual (ML), and distal (D), while in the second, roots were mesial (M), distobuccal (DB), and distolingual (DL). In both configurations, the most common Vertucci classification type was Type I. Types IV and V were less common, with Type IV found in mesial and distal roots, and Type V found in the distolingual root. No teeth with root fusion were identified among three-rooted.

### C-shaped canals

C-shaped canals were observed in 5.36% of cases (14 teeth), predominantly in females (13 teeth, 92.86%) compared to males (1 tooth, 7.14%). These canals were found in both single-rooted (10 teeth, 71.43%) and double-rooted (4 teeth, 28.57%) teeth. The only C-shaped canal identified in a male patient was in a single-rooted tooth.

### Number and distribution of canal orifices

The number and location of canal orifices were analysed in relation to root number. In single-rooted mandibular third molars, one orifice was most frequently observed, although two and three orifices were also present. In two-rooted teeth, the number of orifices most commonly corresponded to the number of roots, with occasional additional canals in the mesial root. In three-rooted molars, a single canal per root predominated, with minimal variation.

**Fig. 2:**
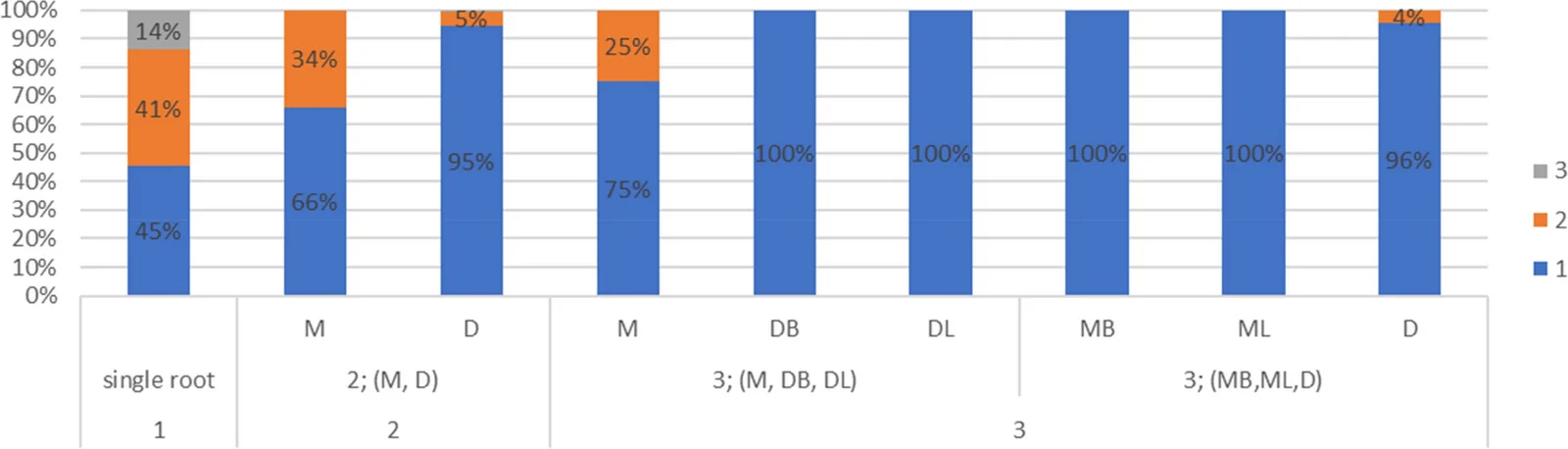
Predicted number of orifices based on root number and position

A detailed distribution of canal orifices according to root number and anatomical location is presented in Fig. 2.

## Discussion

This CBCT-based study aimed to provide comprehensive evaluation of mandibular third molar morphology, with particular focus on identifying the most common configurations in a Central European population, from Wrocław, which is situated in Lower Silesia, Poland (PL5 region under the Nomenclature of Territorial Units for Statistics (NUTS)). Mandibular third molars (MTMs) often present considerable anatomical variability, making them a frequent source of clinical challenges in endodontic practice. To avoid procedural complications during diagnosis and planning, and to ensure successful treatment, thorough understanding of their morphology is essential. Precise evaluation of this complex anatomy requires accurate imaging techniques that allow detailed visualization of root canal systems in vivo. Clearing and staining have historically been considered the gold standard, but CBCT has shown comparable accuracy for assessment of root canal morphology (Drăgan et al. 2016; Shihab and Mahdee 2021). CBCT also allows for larger sample sizes than staining methods, increasing statistical power and generalizability (Sun et al. 2016). Despite some resolution limitations, CBCT offers a reliable, non-invasive method for root canal analysis and is currently one of the best options for clinical use. Additionally, selection bias cannot be excluded, as the study sample consisted of patients referred for CBCT imaging for clinical indications.

In the present study, mandibular third molars mostly presented with two-rooted configuration (79.7%), followed by three rooted (11.9%) and single rooted (8.43%) forms. Those resluts are coherent with majority of previous studies across the populations (Sidow et al. 2000; Gulabivala et al. 2001, 2002; Sert et al. 2011; Kuzekanani et al. 2012; Ahmad et al. 2016; Singh et al. 2020; Al-Qudah et al. 2023). No four rooted forms were found in our study, although in other studies they have been identified.

Comparative analysis suggests population-related differences. The prevalence of single-rooted teeth in the present study was lower than in Turkish and Iranian populations, but higher than in Burmese samples. Similarly, the frequency of three-rooted molars was closer to values reported in Indian populations and higher than in several earlier studies. These differences may be attributed to ethnic differences, methodological variation, or sample characteristics (Sidow et al. 2000; Singh et al. 2020).

Sex related differences were also noted. Two rooted teeth were more prevalent in males (87.3%) than in females (74.2%), but females showed greater anatomical variability, meaning higher proportion of single- (11.9% vs 3.6%) and three-rooted (13.9% vs 9.1%) teeth. However, not all differences reached statistical significance. These findings differ from some earlier studies (Somasundaram 2017; Jing et al. 2023), which may again reflect methodological differences or characteristics specific to the population. Therefore, it is suggested to consider sex differences in future studies on mandibular third molars. The age distribution of the study population should also be considered when interpreting the results, although no age-based subgroup analyses were performed.

Regarding root canal morphology, Vertucci’s classification (Vertucci et al. 1974) was used due to its widespread acceptance and comparability with existing literature. Although more complex classifications have been introduced (Weine et al. 1969; Sert and Bayirli 2004; Ahmed et al. 2017, 2020), all observed configurations in this study could be adequately classified within Vertucci’s classification.

Among all root types, Type I of root canal was most common, which was consistent with previous studies (Somasundaram 2017; Priyank et al. 2023). In single rooted teeth Type I predominated, followed by type IV and other, less frequent types. In two-rooted MTMs mesial root had either Type I or Type IV canal configuration, while distal roots were consistently represented by Type I canal configurations. Nearly one-third of mesial roots showed a Vertucci Type IV configuration. Therefore, clinicians should expect the possible presence of a second mesial canal during endodontic treatment.

Comparisons with other populations revealed significant differences, especially in the mesial roots of two-rooted molars, where studies from India (Somasundaram 2017; Priyank et al. 2023) and Jordan (Ahmad et al. 2016; Al-Qudah et al. 2023) showed greater variability and a higher prevalence of types II and III. In contrast, the present study showed a more even distribution, dominated by types I and IV.

In three-rooted lower third molars, the type I configuration also predominated, with minor variations. While other studies have reported a more diverse canal system (Ahmad et al. 2016; Somasundaram 2017; Waheed Sharawy and Mohamed Aly Ahmed 2017; Singh et al. 2020; Al-Qudah et al. 2023; Priyank et al. 2023), our findings suggest a relatively simplified morphology in this population.

Mandibular third molars (MTMs) with more than three roots are considered rare, and no such cases were found in the present study. A total of six MTMs with four roots were identified in the previous studies, all of which exhibited Type 1 canal systems. Additionally, two teeth with five roots were reported, presenting a variety of canal configurations. Remarkably there are case reports of successful treatment of such multi-rooted teeth (Plotino 2008; Tomar et al. 2013; Garg et al. 2014; De Bruyn et al. 2020; Jacob et al. 2021).

Root fusion was rare in the present study, occurring in only 0.8% of cases, which is significantly lower than previously reported values (Ahmad et al. 2016; Al-Qudah et al. 2023). This discrepancy may reflect differences in classification criteria or true anatomical variation between populations.

The lack of standardized nomenclature for root anatomy poses an additional challenge. Previous studies have commonly used directional terms such as mesial, distal, and distolingual; however, these classifications did not fully reflect the anatomical patterns observed in our study. Especially, the root distribution in three-rooted molars differed from commonly described configurations. These discrepancies may indicate anatomical variability across populations and highlight the need for more consistent and universal classification systems.

To facilitate a structured comparison with existing literature, additional analyses were compiled in the supplementary materials. A comparison of the prevalence of mandibular third molars with different root numbers across previous studies is presented in Supplement 2, while sex-based distributions are summarized in Supplement 3.

Furthermore, a comprehensive review of relevant studies from the last decade was conducted to contextualize the present findings. Data on root canal configurations, root number, population characteristics, and imaging methodologies were collected and standardized to allow meaningful comparison, and are summarized in Supplement 4 (Gulabivala et al. 2001; Neelakantan et al. 2010; Drăgan et al. 2016; Sun et al. 2016; Somasundaram 2017; Tomaszewska et al. 2018a; Hafezi et al. 2019; Naseri et al. 2019; Shihab and Mahdee 2021; Ahmed 2022).

**Fig. 3:**
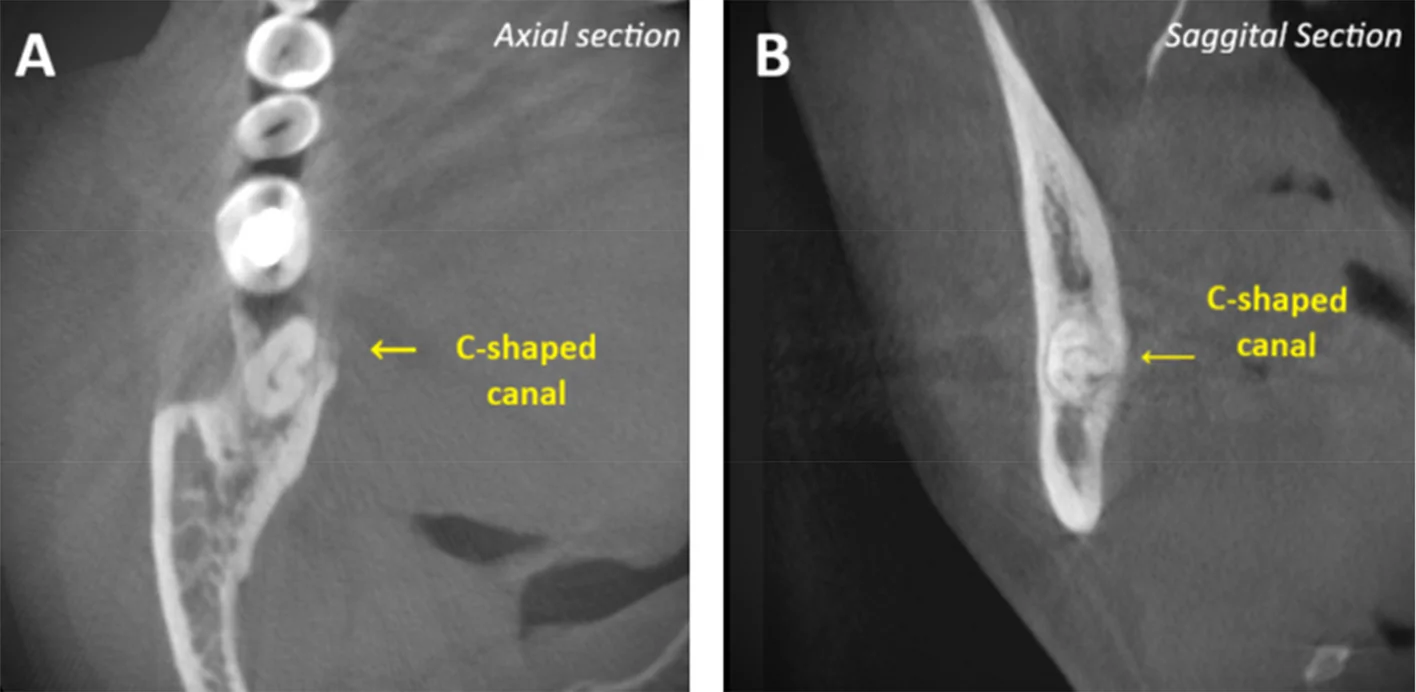
Representative CBCT images of C-shaped canal configurations identified in present study from two different patients. **A** Axial section showing a Type I (continuous) C-shaped canal system. **B** Sagittal section showing a Type III C-shaped canal system

C-shaped canals represent a distinct root canal shape in which, when observed in cross-sectional view, the tooth appears as a C or semi-colon. A semi-continuous C-shaped variation is also possible. The most common cause of this canal formation is the failure of the Hertwig epithelial root sheath to fuse during root development. This configuration was first described by Cooke and Cox in 1979 (Cooke and Cox 1979). The first classification was introduced by Melton in 1991 (Melton et al. 1991), it consisted of three types I – continuous C shape, II – semicolon, III – 2 or 3 separated canals, and it was expanded by Fan in 2004 (Fan et al. 2004), who added Type IV – single round or oval canal in C shaped root and type V with no visible lumen or just near the apex. Figure 3 shows the examples of C-shaped canals observed in our patients.

Clinically, C-shaped canals present significant challenges due to their complex anatomy, including fins, webs, and interconnections, which may complicate debridement, shaping, and obturation. This study showed a 5.36% prevalence of C-shaped canals in the mandibular third molars, which when compared with earlier studies is more consistent with more recent studies than with earlier results. Study by Tomaszewska et al. (2018a, b) (Tomaszewska et al. 2018a) reported a markedly lower prevalence with only 0.8% of cases in her study. Newer study by Somasundaram et al. (2017) (Somasundaram 2017) presented a 10.3% prevalence, predominantly in single-rooted MTMs —a pattern that closely matches the present study’s observation that over two-thirds of C-shaped canals occurred in single-rooted teeth. Furthermore, Priyank et al. (Priyank et al. 2023) reported a slightly higher prevalence of C-shaped canals (7.58%) than in the current study. Present study showed a strong female predominance (92.86%) which has not been underlined in earlier studies, suggesting that future research may benefit from investigating the sex-related factors in C-shaped canal formation.

These results suggest that the prevalence in the present study is higher than that reported in older data, but remains within the range of more recent observations, despite the ethnic differences between the Indian population studies conducted by Somasundaram (Somasundaram 2017) and Priyank (Priyank et al. 2023) and the Polish population studies in the present study. Taken together, these results reflect the evolving understanding of the prevalence and complexity of C-shaped canal morphology in mandibular third molars and emphasize the importance of both sample characteristics and diagnostic methodologies in estimating the prevalence.

The predominance of C-shaped canals in single-rooted mandibular third molars suggests that external root morphology may serve as a useful clinical indicator of internal canal complexity. Consequently, CBCT assessment should be considered before endodontic treatment of single-rooted mandibular third molars whenever complex canal anatomy is suspected.

It is relevant to mention that the term “C-shaped” was used by the authors to describe the shape of the canal, but it can also refer to the shape of the root. For example, Al-Qudah et al. (Al-Qudah et al. 2023) used the term “C-shaped” to define a subtype of single-rooted teeth, distinguishing it from another type they describe as “conical.” In contrast, Ahmad et al. (2016) (Ahmad et al. 2016) and Park et al. (2013) (Park et al. 2013) use the term “C-shaped root” to describe teeth regardless of the number of roots.

**Fig. 4:**
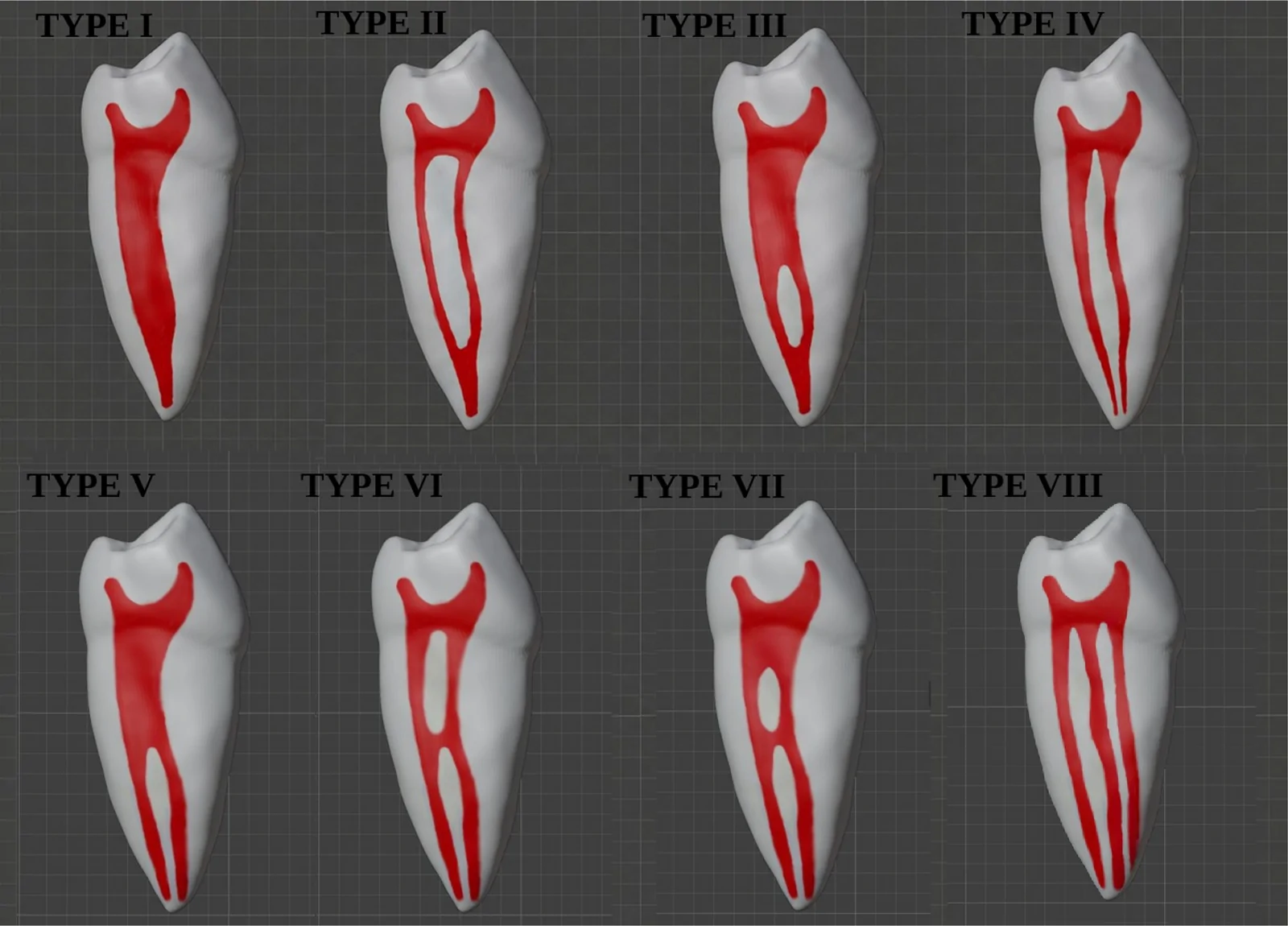
Schematic representation of root canal configurations according to Vertucci’s classification (Types I–VIII)

The findings of this study have important clinical implications. In routine dental treatment, endodontists typically rely on 2D x-radiographs, which do not allow for detailed visualization of root canal anatomy. In such cases, the results of the present study provide valuable guidance, enabling clinicians to make informed predictions about potential canal configurations based on root number, as illustrated in Fig. 4. In those cases, knowledge of relationship between root number and probable canal configuration may support clinical decisions and reduce the risk of missing the canal.

Clinically, the predominance of Type I canal configurations may simplify treatment planning in most mandibular third molars. However, the relatively high prevalence of C-shaped canals and the greater anatomical variability observed in single-rooted teeth shows the importance of careful radiographic assessment and awareness of possible anatomical variations. The anatomical complexity should always be considered during treatment planning. Apart of clinical application, this study allows for a better understanding of population-specific anatomical variability and shows the importance of training in treatment of complex root canal systems, like C-shaped canals.

Among the limitations of our study, we must state that the sample size was limited, and the study population was chosen exclusively from the Lower Silesia region, which may not be a representative for broader populations. We could not use the small field of view CBCT images (5 × 5 cm) due to the limited number of cases, although they might have provided additional information about root canal anatomy. A small field of view is generally used by endodontists, but in our facility, full CBCT scans—or maxillary or mandibular CBCT scans—remain the most commonly performed. Regional genetic, and environmental factors could influence MTM morphology, hence limiting the generalizability of the findings to other populations. Given the single-region sample and specific demographic characteristics of the study group, the applicability of our results to broader populations or different clinical contexts should therefore be considered with caution. Although this study utilized CBCT for accurate assessment of root and canal morphology, its application in clinical practice is limited. Endodontists often depend on conventional periapical radiographs, which do not provide the same level of detail as CBCT scans. Consequently, translating the detailed anatomical findings from this study to routine endodontic practice may be challenging, as clinicians must infer 3D structures from 2D images. This discrepancy may affect the precision and the result of endodontic treatment.

A significant limitation affecting this and other anatomical studies is the absence of a standardization on the terminology and classification of root and canal anatomy. Variations in defining terms such as “separate roots”, "root bifurcation", and “root fusion” can make the direct comparisons between studies difficult. Since the study relied on the availability of CBCT scans, the sample may be biased toward individuals requiring imaging for clinical purposes (e.g., orthodontic treatment or oral surgery, or endodontic treatment). This may accidentally exclude individuals with no clinical need for CBCT imaging, thereby decreasing the representativeness of the sample.

AI-assisted CBCT segmentation represents a promising direction for future anatomical studies. It facilitates use of larger datasets and improves reproducibility through reduced observer variability.

Overall, the findings of this study should be interpreted with caution. While the results confirm previously reported patterns of mandibular third molar morphology and provide new data for a Central European population, the limited sample size, single-region recruitment, and absence of sensitivity analyses restrict the generalizability of the conclusions. Taken together with the variability observed in other populations and across different methodologies, our results underscore the need for careful consideration when extrapolating these findings to broader clinical or research contexts.

Major strengths of this study include standardized CBCT acquisition parameters, calibration of two experienced observers, assessment of inter- and intraobserver agreement, adherence to STROBE and AQUA recommendations, and reporting of confidence intervals.

This study represents one of the largest CBCT investigations of mandibular third molars conducted in a Central European population and one of the few studies simultaneously evaluating root number, Vertucci canal configuration, C-shaped canals, sex differences, and canal orifice distribution using a standardized imaging protocol. By providing standardized anatomical data from a Polish population, it also helps address the current shortage of comparable European CBCT studies on mandibular third molars.

## Conclusions

This CBCT-based study provides new insight into the complex root and canal morphology of mandibular third molars (MTMs) in a Central European population. It expands the limited anatomical data available for that region and provides clinically relevant information regarding the prediction of root canal morphology in mandibular third molars.

Two-rooted configurations were most common, regardless of sex, with clear differences in root morphology observed between sexes. Vertucci’s Type I canal system predominated across all root types, while C-shaped canals—though relatively uncommon—were significantly more frequent in female subjects and single-rooted MTMs.

These findings have important clinical implications. They can assist endodontists in anticipating root canal anatomy based on root number and patient demographics, especially when CBCT is unavailable and clinicians must rely on 2D radiographs. The study also highlights the anatomical variability among populations and the need for tailored diagnostic approaches.

Future research should aim to improve population diversity in anatomical studies, adopt consistent terminology, and explore the integration of AI-assisted CBCT analysis to enhance accuracy, efficiency, and reproducibility in root canal morphology assessment.

*******Ethics approval and consent to participate

We hereby confirm that our work fully complies with the ethical requirements and policies specified by the journal. All research has been conducted in accordance with recognized ethical standards, and no part of the study violates the principles of integrity, transparency, or responsible authorship. All authors have contributed to the work, approved the final version of the manuscript, and agree with its submission. The Bioethics Committee of the Wrocław Medical University approved the study protocol in accordance with the Declaration of Helsinki (approval no. KB 206/2022).

******Declaration of generative AI and AI-assisted technologies in the writing process

During the preparation of this work the author(s) used OpenAi chatGPT in order to improve the readability and language of the manuscript and as an assisting tool during extraction and parsing of tabular data from other sources. After using this tool/service, the author(s) reviewed and edited the content as needed and take(s) full responsibility for the content of the published article.

Supplementary Material 1: AQUA Checklist.

Supplementary Material 2: Prevalence of mandibular third molars with different root numbers in previous studies and the present one. Data adapted from Al-Qudah et al. 2023 licensed under Creative Commons Attribution 4.0 International License.

Supplementary Material 3: Sex-based distribution of MTMs in the present and previous studies.

Supplementary Material 4: Root canal configurations of MTMs with Different Root Numbers in the Present Study and Previous Studies.

Supplementary Material 5: Data supporting study, according to data availability statement.

Supplementary Material 6: STROBE (Strengthening the Reporting of Observational Studies in Epidemiology) Checklist.

## Supplementary Information

Additional file1 (DOCX 35 KB)

Additional file2 (DOCX 16 KB)

Additional file3 (DOCX 37 KB)

Additional file4 (XLSX 24 KB)

Additional file5 (XLSX 74 KB)

Additional file6 (DOCX 43 KB)

## Data Availability

The data that support the findings of this study are available in Supplementary files under Supplement 5.
