# Supplementary material for: Morphology of third mandibular molars – an in vivo cone beam computed tomography study in Lower Silesia
Source: BMC Oral Health. 2026 Aug 13;26:2243. doi: 10.1186/s12903-026-09559-x (PMC13757109; doi:10.1186/s12903-026-09559-x)
Supplement: Supplementary file 1 [file 12903_2026_9559_MOESM1_ESM.docx]

**Anatomical Quality Assurance (AQUA) Checklist**

*“For improving the quality and reporting of anatomical studies.”*

| **Checklist Component** | **#** | **Description and Recommendation:** | **Page Number*** |
| --- | --- | --- | --- |
| **Title** | | | |
| Title | 1 | Identify the main objective or key characteristic of the study in the title. | 1 |
| **Abstract** | | | |
| Structured Summary | 2 | Provide a clear and structured summary of the study with emphasis on the aims, methodology, key findings, and conclusions directly supported by study findings. | 1 |
| **Introduction** | | | |
| Background / Rationale | 3 | Provide a rationale for the study including a concise, updated scientific background, appropriately referenced. Identify any relevant knowledge gaps to support the study rationale. | 2,3 |
| Objective | 4 | Indicate clearly the main objective(s) of the study, and state any hypotheses to be tested. | 2,3 |
| **Methodology** | | | |
| Study Design and Fundamentals | 5 | Provide precise details with respect to the design and fundamentals of the study, including but not limited to the following:   1. Study design: prospective, retrospective, cross-sectional, etc. 2. Study type: cadaveric (e.g. formalin fixed or fresh frozen), imaging, intraoperative, etc. | 3 |
| Setting | 6 | Describe clearly the location where the study was conducted and dates (month/year) between which the data were collected. | 3 |
| Sample Size | 7 | When appropriate, statistical power analysis should be used to calculate sample size or effect size. If relevant, justification for the study sample size should be briefly stated. | 3 |
| Subjects | 8 | Define clearly the eligibility criteria and methods of subject selection and inclusion, with details of the baseline and demographic selection criteria of the subjects (age, sex, healthy or diseased etc.) included in the study. | 3,4 |
| Reference Standard | 9 | Define clearly and accurately all anatomical definitions (normal anatomy, variations, classifications, etc.) by which data will be collected, analyzed, and compared. Citations should be included when appropriate. | 4 |
| Outcomes and/or Parameters | 10 | Define clearly the outcomes and parameters (e.g. prevalence of a variation, mean length and diameter of a structure, etc.) assessed in the study. When present, confounders should be clearly stated. | 3 |
| Measurement and Assessment | 11 | Indicate clearly the group of subjects included in each measurement/assessment (source of data).  Provide clear details about the methods of measurement/assessment of each outcome and/or parameter (e.g. reference points for length measurements, internal or external diameter, etc.). | 3, 4 |
| **Modality** | 12 | Describe clearly the materials, equipment, and instruments used (with manufacturer/supplier details) to conduct the specific study design. | 3 |
| Technique | 13 | Describe precisely the methods (e.g. dissection technique, image reconstruction, etc.) applied in the study to allow for reproducibility. Relevant details (profession, years of experience) regarding the individual(s) performing the technical aspect of the study are recommended. | 3  *Page 1 of 2* |
| Bias | 14 | Identify any potential source of bias and, when present, describe measures implemented to assess the risk of bias. | 17/18 |
| Statistical Approach | 15 | Describe all statistical methods for analyzing the data, including those of confounders. Statistical methods for additional analyses (e.g. subgroup/sensitivity analyses), when performed, should be described. | 5 |
| Ethics | 16 | Provide the details of compliance with ethical guidelines, including the name of the review board or agency, approval number, and date. AQUA endorses the Helsinki Declaration and its later amendments. When appropriate, details of written, informed consent should be clearly stated. | 3 |
| **Results** | | | |
| Subjects | 17 | Report the numbers of subjects included in the study, including data on their baseline and demographic characteristics. When needed, provide reason(s) and data on characteristics of the subjects excluded from the study at any stage. | 4 |
| Main Results | 18 | Provide unaltered/non-manipulated summary data (number [percentage]) or estimates (with confidence intervals and values of consistency when applicable) from the analyses performed. Tabular presentation of the results is highly recommended. | 5,6,7,8,9,10,11 |
| Descriptive Anatomy | 19 | Present clear and comprehensible figures (i.e. images, illustrations, diagrams, etc.), labeled as appropriate, to explain the results where needed AND describe clearly any anatomical findings that could be ambiguous, questionable, or atypical. New classifications of anatomical variations should be complemented by representative figures and corresponding dissection/imaging photographs. | 5,6,7,8,9,10,11 |
| Confounders | 20 | Present precise data from assessment/measurement of confounders, if any. | N/A |
| Additional analyses | 21 | Provide clear results of additional analyses (e.g. subgroup/sensitivity analyses), if performed. Tabular presentation of the results is highly recommended. | 14 |
| **Discussion** | | | |
| Key Findings | 22 | Include summary of key evidence/findings from the study pertaining to the rationale/objectives of the study. No new study results should be presented in the discussion. | 11,12,13,14,15,16 |
| Interpretation and Comparison(s) | 23 | Provide comprehensive (but judicious) interpretation of the results from the study, and comparison and/or reference to the results from other studies on the topic, appropriately cited. Meaningful clinical impact/significance of the findings from the study should be discussed where relevant. | 11,12,13,14,15,16 |
| Implication(s) | 24 | State briefly the implications of the findings or potential areas of the study that require further research. | 17 |
| Limitation(s) | 25 | Discuss briefly limitations of the study at any stage, including risk of bias, potential confounders, or intraobserver and/or interobserver variability. | 17, 18 |
| **Conclusions** | | | |
| Summary | 26 | Summarize the key information (i.e. “take-home message”) directly supported by the findings/evidence from the study. | 19 |
| **Other Information** | | | |
| Acknowledgement | 27 | Acknowledge individual(s), institution(s), or third parties who significantly contributed to the study. | 20 |
| Conflict of interest | 28 | Disclose any conflicts of interests related to the study for all contributing authors. | 19 |
| Funding | 29 | Describe sources of funding for the study and any other support. | 19 |

** If an item is not applicable to the study, mark N/A in the page number box.*

*Page 2 of 2*

**Tomaszewski KA, Henry BM, Ramakrishnan PK, et al. Development of the Anatomical Quality Assurance (AQUA) Checklist: Guidelines for Reporting Original Anatomical Studies. Clin Anat. 2016; 30: 14–20. doi:10.1002/ca.22800**

*© International Evidence-Based Anatomy Working Group, Krakow, Poland*
