# Supplementary material for: Morphology of third mandibular molars – an in vivo cone beam computed tomography study in Lower Silesia
Source: BMC Oral Health. 2026 Aug 13;26:2243. doi: 10.1186/s12903-026-09559-x (PMC13757109; doi:10.1186/s12903-026-09559-x)
Supplement: Supplementary file 2 [file 12903_2026_9559_MOESM2_ESM.docx]

| Study | Nationality | Sample size | 1-root (%) | 2-roots (%) | 3-roots (%) | 4-roots (%) |
| --- | --- | --- | --- | --- | --- | --- |
| Sidow et al. | American | 150 | 17 | 77 | 5 | 1 |
| Gulabivala et al. | Burmese | 58 | - | 100 | - | - |
| Gulabivala et al. | Thai | 173 | 12 | 86.8 | 1 | 1 |
| Sert et al. | Turkish | 370 | 24.8 | 69.2 | 5.4 | 0.27 |
| Kuzekanani et al. | Iranian | 150 | 21 | 73 | 5.5 | 0.5 |
| Ahmad et al. | Jordanian | 70 | 12.9 | 74.3 | 8.7 | 4.3 |
| Singh et al. | Indian | 100 | 15 | 63 | 18 | 4 |
| (Al-Qudah et al. 2023) | Jordanian | 639 | 2.5 | 89.7 | 7.3 | 0.47 |
| Olczyk et al. | Polish | 261 | 8.43 | 79.7 | 11.9 | - |

Supplement 2: Prevalence of mandibular third molars with different root numbers in previous studies and the present one. Data adapted from Al-Qudah et al., 2023 licensed under Creative Commons Attribution 4.0 International License

Data were extracted from published studies and harmonized for comparison.
