# Supplementary material for: Morphology of third mandibular molars – an in vivo cone beam computed tomography study in Lower Silesia
Source: BMC Oral Health. 2026 Aug 13;26:2243. doi: 10.1186/s12903-026-09559-x (PMC13757109; doi:10.1186/s12903-026-09559-x)
Supplement: Supplementary file 3 [file 12903_2026_9559_MOESM3_ESM.docx]

| Number of roots | Study | Female (%) | Male (%) |
| --- | --- | --- | --- |
| 1 | Present study | 11.9 | 3.6 |
|  | Jing et al., 2023 | 23.6 | 13.1 |
|  | Somasundaram, 2017 | 0.9 | 0.1 |
| 2 | Present study | 74.2 | 87.3 |
|  | Jing et al.. | 69.1 | 78.9 |
|  | Somasundaram et al.. | 0.3 | 0.7 |
| 3 | Present study | 13.9 | 9.1 |
|  | Jing et al.. | 6.8 | 7.9 |
|  | Somasundaram et al.. | 0.5 | 0.5 |
| 4 | Present study | 0 | 0 |
|  | Jing et al.. | 0.5 | 0.1 |
|  | Somasundaram et al.. | 0 | 0 |
| 5 | Present study | 0 | 0 |
|  | Jing et al.. | 0.5 | 0.1 |
|  | Somasundaram et al.. | 0 | 1 |

Supplement 3 Sex-Based Distribution of MTMs with Different Root Numbers in the Present Study and Previous Studies

Bibliography

Jing, Q., Song, H., Huang, H., Shi, Y., Cheng, J., & Wang, D. (2023). Characterizations of three-dimensional root morphology and topological location of mandibular third molars by cone-beam computed tomography. *Surgical and Radiologic Anatomy*, *45*(5), 527–536. https://doi.org/10.1007/s00276-023-03111-0

Somasundaram, P. (2017). Retrospective Study of Root Canal Configurations of Mandibular Third Molars Using CBCT- Part-II. *JOURNAL OF CLINICAL AND DIAGNOSTIC RESEARCH*, *11*(6), ZC55–ZC59. https://doi.org/10.7860/JCDR/2017/20153.10072
