# Supplementary material for: Morphology of third mandibular molars – an in vivo cone beam computed tomography study in Lower Silesia
Source: BMC Oral Health. 2026 Aug 13;26:2243. doi: 10.1186/s12903-026-09559-x (PMC13757109; doi:10.1186/s12903-026-09559-x)
Supplement: Supplementary file 4 [file 12903_2026_9559_MOESM6_ESM.docx]

STROBE Statement—checklist of items that should be included in reports of observational studies

|  | Item No. | Recommendation | Page  No. | Relevant text from manuscript |
| --- | --- | --- | --- | --- |
| **Title and abstract** | 1 | (*a*) Indicate the study’s design with a commonly used term in the title or the abstract | 3 | This cross-sectional study analysed CBCT scans of 261 mandibular third molars (...). |
|  |  | (*b*) Provide in the abstract an informative and balanced summary of what was done and what was found | 3 | This cross-sectional study analysed CBCT scans of 261 mandibular third molars (....). Most MTMs had two roots (79.69%), followed by three roots (11.81%) and a single root (8.43%). Two-rooted teeth were more frequent in males (87.27%) than females (74.17%, p = 0.027). Type I was the most common root canal configuration in all root categories. C-shaped canals were present in 5.36% of teeth, predominantly in single-rooted teeth (71.43%) and females (92.86%). (...) Findings underscore the prevalence of C-shaped canals and gender-related differences in root structure. |
| Introduction | | | |  |
| Background/rationale | 2 | Explain the scientific background and rationale for the investigation being reported | 4 | Mandibular third molars (MTMs), commonly referred to as wisdom teeth, exhibit significant anatomical variability, posing challenges for dental practitioners in endodontic and surgical procedures. Accurate knowledge of root canal morphology is essential for effective treatment planning and achieving successful clinical outcomes.”  “Although numerous studies have investigated the anatomy of mandibular first… and second molars…, research focusing specifically on mandibular third molars remains limited… Notably, to date, only one study has examined MTMs in the Polish population.”  “Current research on root canal configurations on MTMs reveals several gaps. Many studies lack population diversity… Additionally, the absence of standardized terminology and classification systems makes direct comparisons across studies difficult.”  “The use of cone-beam computed tomography (CBCT) in this study is well-justified based mainly on its balance of accuracy, accessibility, and clinical applicability… CBCT offers a reliable, non-invasive method for root canal analysis and is currently one of the best options for clinical use.” |
| Objectives | 3 | State specific objectives, including any prespecified hypotheses | 4,5 | “In this study, the root canal morphology of the MTM was investigated in a population from Lower Silesia using CBCT imaging. The research question was what is the most common configuration of the root canal system in the mandibular third molars (MTM).”  “This study investigates the root canal morphology of MTMs in a population from Lower Silesia using CBCT imaging. Specifically, it addresses the following questions:  1. How many roots are most commonly present in mandibular third molars (MTMs)?  2. Are there significant differences in the number of roots in MTMs between males and females?  3. What are the most common root canal configurations in mandibular third molars?  4. What is the prevalence of C-shaped root canals in MTMs?”* |
| Methods | | | |  |
| Study design | 4 | Present key elements of study design early in the paper | 5,6 | This cross-sectional study analysed CBCT scans of 261 mandibular third molars from patients aged 18 and over were analysed according to Vertucci’s classification. Data collection took place at the X-Ray Diagnostic Laboratory of (City name anonymised due to double blind policy) Medical University from January to April 2022. |
| Setting | 5 | Describe the setting, locations, and relevant dates, including periods of recruitment, exposure, follow-up, and data collection | 5,6 | The CBCT scans were obtained from the Academic Dental Polyclinic of (City name anonymised due to double blind policy) Medical University in Poland, specifically from the X-Ray Diagnostic Laboratory.” / “These scans were conducted… between January and April 2022 using a Rayscan α-Plus 130 (Rayscan 700) CBCT scanner…” / “Research access to the data was granted on June 7, 2022.” / “Assessments were performed in a dimly lit room, with no more than 15 scans evaluated per session to minimize fatigue. |
| Participants | 6 | ~~(~~*~~a~~*~~)~~ *~~Cohort study~~*~~—Give the eligibility criteria, and the sources and methods of selection of participants. Describe methods of follow-up~~  *~~Case-control study~~*~~—Give the eligibility criteria, and the sources and methods of case ascertainment and control selection. Give the rationale for the choice of cases and controls~~  *Cross-sectional study*—Give the eligibility criteria, and the sources and methods of selection of participants | 5,6 | The CBCT scans were obtained from the Academic Dental Polyclinic of (City name anonymised due to double blind policy) Medical University in Poland…” / “All available scans (n = 316) were analysed… 261 mandibular third molars met the inclusion criteria.” / Standardization of included scans: “To standardize the study, full-mouth CBCT scans with a voxel size of 160 µm, slice thickness of 0.16 mm, field of view of 13x10 cm, and settings of 90 kV and 4 mA were used.” / Eligibility: “Inclusion criteria were: participants aged 18 or older; teeth without caries, fillings, prosthetic crowns, or post-core inlays; teeth without previous endodontic treatment; teeth with closed apices, free from resorptions or calcifications; and artifact-free CBCT images.  CBCT scans were obtained for orthodontic, surgical, and endodontic indications. |
|  |  | ~~(~~*~~b~~*~~)~~ *~~Cohort study~~*~~—For matched studies, give matching criteria and number of exposed and unexposed~~  *~~Case-control study~~*~~—For matched studies, give matching criteria and the number of controls per case~~ |  |  |
| Variables | 7 | Clearly define all outcomes, exposures, predictors, potential confounders, and effect modifiers. Give diagnostic criteria, if applicable | 5, 6, 7 | The number of roots, root canals, and root canal configurations were assessed according to Vertucci's classification (1974) … Vertucci's classification delineates root canal configurations as follows: Type I … Type VIII …” • “Inclusion criteria were: participants aged 18 or older; teeth without caries, fillings, prosthetic crowns, or post-core inlays; teeth without previous endodontic treatment; teeth with closed apices, free from resorptions or calcifications; and artifact-free CBCT images C-shaped canals were identified based on axial morphology; root fusion was defined as lack of clear separation between roots (Methods). |
| Data sources/ measurement | 8* | For each variable of interest, give sources of data and details of methods of assessment (measurement). Describe comparability of assessment methods if there is more than one group | 5,6 | The CBCT scans were obtained from the Academic Dental Polyclinic of (City name anonymised due to double blind policy) Medical University … between January and April 2022 using a Rayscan α-Plus 130 (Rayscan 700) CBCT scanner … field of view … 60–90 kV, 4–17 mA … voxel sizes … slice thickness …” • “To standardize the study, full-mouth CBCT scans with a voxel size of 160 µm, slice thickness of 0.16 mm, field of view of 13x10 cm, and settings of 90 kV and 4 mA were used. Data were reviewed using Rayscan SMART Dent … and OnDemand3DApp …” • “The images were evaluated by two endodontists … Disagreements were resolved through consensus. … Assessments were performed in a dimly lit room, with no more than 15 scans evaluated per session to minimize fatigue.” • “Before evaluating the CBCT scans, both endodontists underwent training and calibration … Cohen's kappa … 91.7%. |
| Bias | 9 | Describe any efforts to address potential sources of bias | 3, 5, 6 | To standardize the study, full-mouth CBCT scans … were used.” • “Before evaluating the CBCT scans, both endodontists underwent training and calibration … Cohen's kappa … 91.7%, indicating excellent agreement.” • “Assessments were performed … with no more than 15 scans evaluated per session to minimize fatigue.” • “Inclusion criteria … artifact-free CBCT images.” Additionally, selection bias cannot be excluded, as the study sample consisted of patients referred for CBCT imaging for clinical indications (Discussion – limitations).  Observer bias was minimized through calibration and consensus evaluation. |
| Study size | 10 | Explain how the study size was arrived at | 7 | All available scans (n = 316) were analysed, and the power of the analysis … (n = 261) was calculated afterwards … a sample size of 261 allows for the detection of Cohen’s effect size “w” of 0.172 with a power of 0.64 and an alpha level of 0.05.  No a priori sample size calculation was performed due to the retrospective design. |

Continued on next page

| Quantitative variables | 11 | Explain how quantitative variables were handled in the analyses. If applicable, describe which groupings were chosen and why | 6 | Inclusion criteria were: participants aged 18 or older (continous variable, with no further categorization); (...)  The number of roots, root canals, and root canal configurations were assessed according to Vertucci's classification (1974) (...) Root and root canal number were treated as categorical variables. |
| --- | --- | --- | --- | --- |
| Statistical methods | 12 | (*a*) Describe all statistical methods, including those used to control for confounding | 7 | Table 2 presents chi-squared and p-values … The chi-squared test yielded a significance level below 0.05. The Bonferroni correction was applied to control for Type I error … The threshold for statistical significance was set at 0.05. Confidence intervals …”  Control for confounding was addressed by inclusion criteria, no additional statistical adjustment for confounding was applied. No multivariable analyses were performed as the study was descriptive in nature. |
|  |  | (*b*) Describe any methods used to examine subgroups and interactions | 7 | Table 2 presents chi-squared and p-values comparing the frequency of each tooth type by sex. |
|  |  | (*c*) Explain how missing data were addressed | 7 | Only artifact-free CBCT images were included; therefore, no missing data were present in the analyses. |
|  |  | ~~(~~*~~d~~*~~)~~ *~~Cohort study~~*~~—If applicable, explain how loss to follow-up was addressed~~  *~~Case-control study~~*~~—If applicable, explain how matching of cases and controls was addressed~~  *Cross-sectional study*—If applicable, describe analytical methods taking account of sampling strategy | 7 | All available scans meeting the inclusion criteria were analyzed; no complex sampling strategy was applied, and hence no analytical methods to account for sampling design were required. |
|  |  | (*e*) Describe any sensitivity analyses | 7 | Sensitivity analyses were not applicable in this study. |
| Results | | | | |
| Participants | 13* | (a) Report numbers of individuals at each stage of study—eg numbers potentially eligible, examined for eligibility, confirmed eligible, included in the study, completing follow-up, and analysed | 8 | Figure 1 Exclusion criteria |
|  |  | (b) Give reasons for non-participation at each stage | 8 | Figure 1 Exclusion criteria |
|  |  | (c) Consider use of a flow diagram | 8 | Figure 1 Exclusion criteria |
| Descriptive data | 14* | (a) Give characteristics of study participants (eg demographic, clinical, social) and information on exposures and potential confounders | 8 | These teeth came from 151 females (57.9%) and 110 males (42.1%).” age (mean, SD, median, range), sex distribution, number of patients and teeth (Results, section 3.2) |
|  |  | (b) Indicate number of participants with missing data for each variable of interest | 8 | No missing data were present in the analyses |
|  |  | ~~(c)~~ *~~Cohort study~~*~~—Summarise follow-up time (eg, average and total amount)~~ |  |  |
| Outcome data | 15* | *~~Cohort study~~*~~—Report numbers of outcome events or summary measures over time~~ |  |  |
|  |  | *~~Case-control study—~~*~~Report numbers in each exposure category, or summary measures of exposure~~ |  |  |
|  |  | *Cross-sectional study—*Report numbers of outcome events or summary measures | 8-14 | Out of the 261 teeth scans analysed, two-rooted teeth were the most prevalent, accounting for 79.69% (n=208). Three-rooted teeth … 11.81% (n=31) … single-rooted … 8.43% (n=22)” … “Table 1 Distribution (%) of roots … with 95% confidence intervals (CI)” … “C-shaped canals were observed in 5.36% of cases (14 teeth)…” … “Figure 2 … Table 4 … Table 5 … providing 95% confidence intervals… |
| Main results | 16 | (*a*) Give unadjusted estimates and, if applicable, confounder-adjusted estimates and their precision (eg, 95% confidence interval). Make clear which confounders were adjusted for and why they were included | 14 | Only unadjusted estimates were presented. No confounder-adjusted analyses were performed beyond restriction by inclusion criteria. Precision of estimates was expressed with 95% confidence intervals. |
|  |  | (*b*) Report category boundaries when continuous variables were categorized | 14 | Age was recorded as a continuous variable with an inclusion threshold of 18 years; no further categorization was applied. |
|  |  | (*c*) If relevant, consider translating estimates of relative risk into absolute risk for a meaningful time period | 14 | Relative and absolute risk estimates were not applicable in this morphological study. |

Continued on next page

| Other analyses | 17 | Report other analyses done—eg analyses of subgroups and interactions, and sensitivity analyses | 14 | Subgroup analyses were performed by sex for the number of roots and root canal configurations. Post hoc comparisons with Bonferroni correction were conducted to assess differences between male and female participants. No interaction analyses or sensitivity analyses were performed |
| --- | --- | --- | --- | --- |
| Discussion | | | | |
| Key results | 18 | Summarise key results with reference to study objectives | 15 | The results of the study indicate that two-rooted teeth are the most common in mandibular third molars, while single- and three-rooted configurations occur much less frequently. Differences were observed between men and women in the frequency of root configurations; single-rooted teeth were more common in women, whereas two-rooted teeth were more common in men, although not all differences reached statistical significance. Vertucci type I canals were the most common in all root configurations; however, more complex canal types, including C-shaped canals, were also observed, particularly in single-rooted teeth and female patients. |
| Limitations | 19 | Discuss limitations of the study, taking into account sources of potential bias or imprecision. Discuss both direction and magnitude of any potential bias | 21 | Sample size was limited (...) limited number of cases (...) absence of a standardization on the terminology and classification of root and canal anatomy (...) |
| Interpretation | 20 | Give a cautious overall interpretation of results considering objectives, limitations, multiplicity of analyses, results from similar studies, and other relevant evidence | 15, 16 | These differences could be attributed to differences in demographic characteristics, imaging methods, or sample sizes. Therefore, it is essential to consider sex differences in future studies on mandibular third molars. (…)  Overall, the findings of this study should be interpreted with caution. While the results confirm previously reported patterns of mandibular third molar morphology and provide new data for a Central European population, the limited sample size, single-region recruitment, and absence of sensitivity analyses restrict the generalizability of the conclusions. Taken together with the variability observed in other populations and across different methodologies, our results underscore the need for careful consideration when extrapolating these findings to broader clinical or research contexts. |
| Generalisability | 21 | Discuss the generalisability (external validity) of the study results | 21 | Among the limitations of our study, we must state that the sample size was limited, and the study population was chosen exclusively from the Lower Silesia region, which may not be a representative for broader populations. (...) Regional genetic, and environmental factors could influence MTM morphology, hence limiting the generalizability of the findings to other populations. Given the single-region sample and specific demographic characteristics of the study group, the applicability of our results to broader populations or different clinical contexts should therefore be considered with caution (…) This underscores the importance of including sex as a variable in future research. (…) The generalisability of our results is limited by the single-region sample and specific demographic characteristics of the study population, and therefore the findings should be interpreted with caution when applied to other populations or clinical contexts.  The generalisability of the findings is limited due to single-region sampling and demographic specificity. |
| Other information | |  | | |
| Funding | 22 | Give the source of funding and the role of the funders for the present study and, if applicable, for the original study on which the present article is based | 14 | This study was funded by (City name anonymised due to double blind policy) Medical University as part of the co-financing provided under the subsidy for maintaining and developing research potential in 2022 (SUBZ.B010.22.040). The funding source had no involvement in the study's design, data collection or analysis, decision to publish, or manuscript preparation. No additional external funding was received for this research. |

*Give information separately for cases and controls in case-control studies and, if applicable, for exposed and unexposed groups in cohort and cross-sectional studies.

**Note:** An Explanation and Elaboration article discusses each checklist item and gives methodological background and published examples of transparent reporting. The STROBE checklist is best used in conjunction with this article (freely available on the Web sites of PLoS Medicine at http://www.plosmedicine.org/, Annals of Internal Medicine at http://www.annals.org/, and Epidemiology at http://www.epidem.com/). Information on the STROBE Initiative is available at www.strobe-statement.org.
